# Descriptions of two new and one known species of *Parkellus* Jairajpuri, Tahseen and Choi, 2001 (Nematoda: Mononchidae) and their phylogenetic position among Mononchida

**DOI:** 10.21307/jofnem-2021-076

**Published:** 2021-08-25

**Authors:** Tam T.T. VU, Katarzyna Rybarczyk-Mydłowska, Andrij Susulovsky, Magdalena Kubicz, Łukasz Flis, Thi Mai Linh Le, Grażyna Winiszewska

**Affiliations:** 1 Institute of Ecology and Biological Resources VAST, 18 Hoang Quoc Viet, Cau Giay, Hanoi, Vietnam; 2 Graduate University of Science and Technology VAST, 18 Hoang Quoc Viet, Cau Giay, Hanoi, Vietnam; 3 Museum and Institute of Zoology PAS, Wilcza 64, 00-679 Warsaw, Poland; 4 State Museum of Natural History NASU, Theatralna str. 18, Lviv, 79008, Ukraine

**Keywords:** Identification key, Morphology, Ribosomal DNA, Taxonomy

## Abstract

Two new species of *Parkellus* (Jairajpuri et al., 2001) from Vietnam and a population of *Parkellus zschokkei* (Ahmad and Jairajpuri, 2010; Menzel, 1913) from Ukraine are described, illustrated and their phylogenetic position among the Mononchida is presented. The molecular data (18S and 28S rDNA) are given for the three investigated species – first time for the genus *Parkellus*. *Parkellus hagiangensis* sp. nov. is characterized by a medium-sized buccal cavity, posterior position of the dorsal tooth located below the beginning of the pharynx, males having the ventromedian cuticular pores above and below the excretory pore, short spicules with conical proximal part; females with very faint *pars refringens vaginae* and small teardrop-shaped pieces, short *pars distalis vaginae*, the presence of small ventromedian vulval papillae. *Parkellus tuyenquangensis* sp. nov. is characterized by a medium-sized buccal cavity, posterior position of the dorsal tooth located above the beginning of the pharynx, males having the ventromedian cuticular pores above and below the excretory pore, medium-sized spicules with a cylindrical proximal part, very short lateral guiding pieces, females with very strongly sclerotized *pars refringens vaginae*, medium size teardrop-shaped pieces, short *pars distalis vaginae* thickened at the junction with *pars refringens vaginae*. The newly described species are morphologically most similar to *P. parkus* and *P. zschokkei*. An identification key to *Parkellus* species is presented.

Predatory nematodes play a substantial role in the soil food-web as regulators of other trophic groups: bacterial, fungal, algae, and herbivorous feeders, they are also suitable bioindicators of soil condition (Ahmad and Jairajpuri, 2010; Ferris and Bongers, 2006; Ferris et al., 2001, 2012; Holterman et al., 2008; Moens et al., 2000). Unfortunately, many of the predaceous species which have been described still lack molecular characterization, while a great number might need to be discovered yet.

Herein, the descriptions of two new species of predatory soil nematodes of the genus *Parkellus* are provided. Jairajpuri et al. (2001) established the monotypic genus *Parkellus* for the new species *P. parkus* collected in Korea from forest soil surrounding the roots of *Euonymus oxyphyllus* Miq. The main feature that distinguished the newly described genus *Parkellus* from the morphologically very similar *Coomansus* (Jairajpuri and Khan, 1977) is the location of dorsal tooth in the posterior half of the buccal cavity. Zullini and Peneva (2006) and Andrássy (2009) contested such a conclusion assuming that the occurrence of the above mentioned feature is not sufficient to make such a distinction. In 2010, Ahmad and Jairajpuri published a summary of the knowledge on the mononchid nematodes, in which they presented several changes in the taxonomy of this group of predatory nematodes. One of them was the transfer of nine species in which the dorsal tooth is located in the posterior half of the buccal cavity from genus *Coomansus* to the genus *Parkellus*. Andrássy (2011) did not support the separation of the genus *Parkellus* from the genus *Coomansus* because the distinguishing characters such as posteriorly situated dorsal tooth apex and a complex gubernaculum can be observed also in some species of *Coomansus* and regarded the genus *Parkellus* as a synonym of the genus *Coomansus*. However, Siddiqi (2015) believed that the size and position of the dorsal tooth on the dorsal metarhabdion are of fundamental importance in the classification and phylogeny of Mononchida, as is the occurrence of two subventral teeth and their relative position with respect to the dorsal tooth. He agreed with the separation of the genus *Parkellus* from the genus *Coomansus*. He has separation of several genera based on the position of dorsal tooth apex on dorsal metarhabdion: the new genus *Supronchus* from the genus *Iotonchulus*, *Megaiotonchus* from the genus *Iotonchus* (Iotonchidae), the new genus *Pentonchella* (Anatonchidae) (Siddiqi, 2015).

Of the 10 *Parkellus* species described so far, nine are found in Asia, three in Europe, and one in North America (Ahmad and Jairajpuri, 2010; Choi and Choi, 1997; Holovachov, 2014; Jairajpuri et al., 2001; Vu and Winiszewska, 2020).

In the present study, two new species: *Parkellus hagiangensis* sp. nov. and *Parkellus tuyenquangensis* sp. nov. from Vietnam are described based on morphological, morphometric, and molecular data. Their phylogenetic position among the Mononchida is presented. An updated key to 12 *Parkellus* species is provided.

## Material and methods

### Nematode extraction, preservation, and morphological studies

Soil samples were collected from a pristine forest in Vietnam and from an oak forest in Ukraine. Nematodes were extracted from soil samples using modified Baermann funnel technique (Southey, 1986).

They were heat killed, fixed in 4% formaldehyde (for morphological observations) or in a DESS mixture (Yoder et al., 2006) (for molecular analyses), transferred to anhydrous glycerol (Seinhorst, 1962), and mounted on glass slides for microscopic observation. Measurements were performed with a Nikon digital camera on a Nikon Eclipse Ni microscope at the Institute of Ecology and Biological Resources, Vietnam Academy of Science and Technology (VAST), Vietnam. Observations of morphological diagnostic features were performed in a Leica DM5000B light microscope using the Nomarsky differential interference contrast (DIC) technique. Illustrations were drawn and photographs were taken using a Leica DFC 500 camera on Leica DM5000B microscope at the Museum and Institute of Zoology Polish Academy of Sciences (PAS), Warsaw, Poland. Illustrations were edited by using Adobe Photoshop CC 2018. The spicule terminology is expressed according to Peña-Santiago et al. (2014).

### Nematode lysis, amplification, and sequencing of rDNA fragments

After conducting the microscopic studies and preparing the photographic documentation, all nematodes subjected to molecular analyses were taken out from the temporary slides. Each retrieved nematode was transferred to a separate dish filled with PBS (phosphate buffered saline) solution. The dishes with nematodes were placed on a laboratory shaker. The nematodes were initially washed in PBS for 3  hr. Subsequently, the nematodes were transferred into dishes containing fresh PBS and were washed on a shaker overnight. In the final washing stage, the nematodes were transferred into dishes containing sterile milliQ water and were washed with it for 4 hr. After this procedure, the nematode individuals were transferred to separate 0.2 ml polymerase chain reaction (PCR) tubes containing 25 μl sterile water. An equal volume of the lysis buffer, as described in Holterman et al. (2006), was added to every tube. The lysis occurred in a thermal cycler (Veriti 96*-*Well Thermal Cycler, Applied Biosystems, Foster City, CA, USA) at 65°C for 3 hr followed by a 5 min incubation at 100°C. The obtained single nematode lysate (crude DNA extract) was used either immediately as a DNA template for a PCR reaction or stored at ‒ 20°C for future use.

Nearly full length 18S rDNA was amplified from the analyzed *Parkellus* individuals in two overlapping fragments, using the following primer combinations: 988F combined with 1912R and 1813F combined with 2646R (Holterman et al., 2006). The 28S rDNA sequence was also amplified in two parts using the 61F primer (Holterman et al., 2006) combined with the MCB1R (Dobosz et al., 2013) and the D2A primer combined with the D3B (Nunn, 1992).

All PCR reactions contained 12.5 μl Color Perpetual OptiTaq PCR Master Mix (2x) (EURx, Gdansk, Poland), 1 μl of the forward and reverse primer (5 μM each), the 3 μl DNA template and sterile Milli-Q water to 25 μl of the total volume. All PCR reactions were performed in Veriti 96*-*Well Thermal Cycler (Applied Biosystems, Foster City, CA, USA) as follows: an initial denaturation step at 94°C for 3 min, followed by 40 cycles at 94°C for 30 sec, 54°C (58°C or 62°C in case of D2A-D3B primer combination) for 30 sec and 72°C for 60–70 sec with a final incubation for 7 min at 72°C. Amplicons were visualized under UV illumination after Simply Safe (EURx, Gdansk, Poland) gel staining and gel electrophoresis. Excess dNTPs and unincorporated primers were removed from the PCR product using the enzymatic mixture of Fast Polar-BAP – Thermosensitive Alkaline Phosphatase (EURx, Gdansk, Poland) and Exonuclease I – (EURx, Gdansk, Poland).

After sequencing the obtained *Parkellus* rDNA sequence fragments, as well as ribosomal DNA fragments of *Coomansus parvus* used in this study as a close reference species, were deposited in GenBank under the following accession numbers: MT799665–MT799669 (18S rDNA) and MT799670–MT799673 (28S rDNA).

### Sequence analysis and phylogenetic studies

The newly obtained rDNA sequences were analyzed using the BioEdit program (Hall, 1999: v.7.2.5). The final 18S and 28S rDNA datasets for phylogenetic study included sequences from the three studied *Parkellus,* newly acquired *Coomansus parvus* and available sequences of Mononchida representatives from GenBank. Representatives of Dorylaimida, Mermithida, and Trichinellida were used as an outgroup. The final multiple-sequence alignments consisted of 1,688 overlapping characters in case of 18S rDNA and 1,047 characters in case of 28S rDNA. Genetic distances were estimated with the DNADist tool implemented in the BioEdit, using the default options (Kimura 2-parameter model of nucleotide substitution).

The Bayesian phylogenies were constructed with the program MrBayes v. 3.1 (Ronquist and Huelsenbeck, 2003). GTR substitution model with a proportion of invariable sites and gamma distribution for both 18S and 28S data sets was used. Two independent runs were performed with four Markov chains per run. The program was run for 2 × 10^6^ generations in case of the 18S rDNA data set and for 1 × 10^6^ generations in case of the 28S rDNA data set. Sample frequency in both cases was 100 generations. The sampled trees from each run were combined in a single 50% majority-rule tree. Stabilization of the likelihood and parameters was checked with the program Tracer (Rambaut et al., 2014: v.1,6).

## Description and discussion

Systematics

Order Mononchida (Jairajpuri, 1969)

Suborder Mononchina (Kirjanova and Krall, 1969)

Superfamily Mononchoidea (Filipjev, 1934)

Family Mononchidae (Filipjev, 1934)

Genus *Parkellus* (Jairajpuri et al., 2001)

### Type species

*Parkellus parkus* (Jairajpuri et al., 2001)

### Valid species

*Parkellus acuticaudatus* (Eroshenko, 1975; Jairajpuri et al., 2001)

*Iotonchus acuticaudatus* (Eroshenko, 1975)

*Coomansus acuticaudatus* (Eroshenko, 1975; Loof and Winiszewska-Ślipińska, 1993)

*Parkellus cobbi* (Eroshenko, 1975; Jairajpuri et al., 2001)

*Iotonchus cobbi* (Eroshenko, 1975)

*Coomansus cobbi* (Eroshenko, 1975; Loof and Winiszewska-Ślipińska, 1993)

*Parkellus hagiangensis* sp. nov.

*Parkellus menzeli* (Jairajpuri et al., 2001; Loof and Winiszewska-Ślipińska, 1993)

*Coomansus menzeli* (Loof and Winiszewska-Ślipińska, 1993)

*Parkellus monticola* (Eroshenko, 1975; Jairajpuri et al., 2001)

*Iotonchus monticola* (Eroshenko, 1975)

*Coomansus monticola* (Eroshenko, 1975; Loof and Winiszewska-Ślipińska, 1993)

*Parkellus mucronatus* (Eroshenko, 1975; Jairajpuri et al., 2001)

*Iotonchus mucronatus* (Eroshenko, 1975)

*Coomansus mucronatus* (Eroshenko, 1975; Loof and Winiszewska-Ślipińska, 1993)

*Parkellus paraamphigonicus* (Eroshenko, 1975; Jairajpuri et al., 2001)

*Iotonchus paraamphigonicus* (Eroshenko, 1975)

*Coomansus paraamphigonicus* (Eroshenko, 1975; Loof and Winiszewska-Ślipińska, 1993)

*Parkellus silvius* (Eroshenko, 1975; Jairajpuri et al., 2001)

*Clarkus silvius* (Eroshenko, 1975)

*Coomansus silvius* (Eroshenko, 1975; Jairajpuri and Khan, 1982)

*Parkellus simmenensis* (Jairajpuri et al.,2001; Kreis, 1924)

*Mononchus simmenensis* (Kreis, 1924)

*Iotonchus simmenensis* (Andrássy, 1958; Kreis, 1924)

*Coomansus simmenensis* (Kreis, 1924; Loof and Winiszewska-Ślipińska, 1993)

*Parkellus tuyenquangensis* sp. nov.

*Parkellus zschokkei* (Jairajpuri et al., 2001; Menzel, 1913)

*Mononchus zschokkei* (Menzel, 1913)

*Iotonchus zschokkei* (Altherr, 1950; Menzel, 1913)

*Coomansus zschokkei* (Loof and Winiszewska-Ślipińska, 1993; Menzel, 1913)

*Clarkus scopulosus* (Eroshenko, 1975)

*Coomansus scopulosus* (Eroshenko, 1975; Jairajpuri and Khan, 1982)

**Figure 1:**
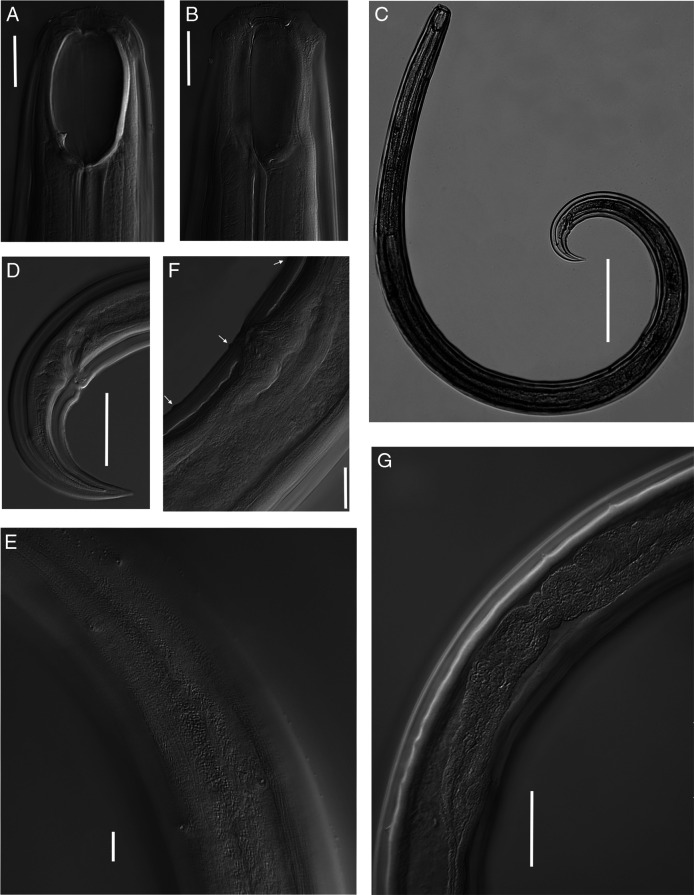
Light micrographs of *Parkellus hagiangensis* sp. nov. Female. A. Anterior end, median section with the focus on the dorsal tooth. B. Anterior end, surface view. C. Entire body. D. Tail. E. Vulval region with advulval ventromedian vulval papillae. F. Body pores. G. Anterior genital branch. Scale bar: C = 200 µm, G = 50 µm, D = 45 µm, A, B, E = 20 µm, F = 10 µm.

**Figure 2:**
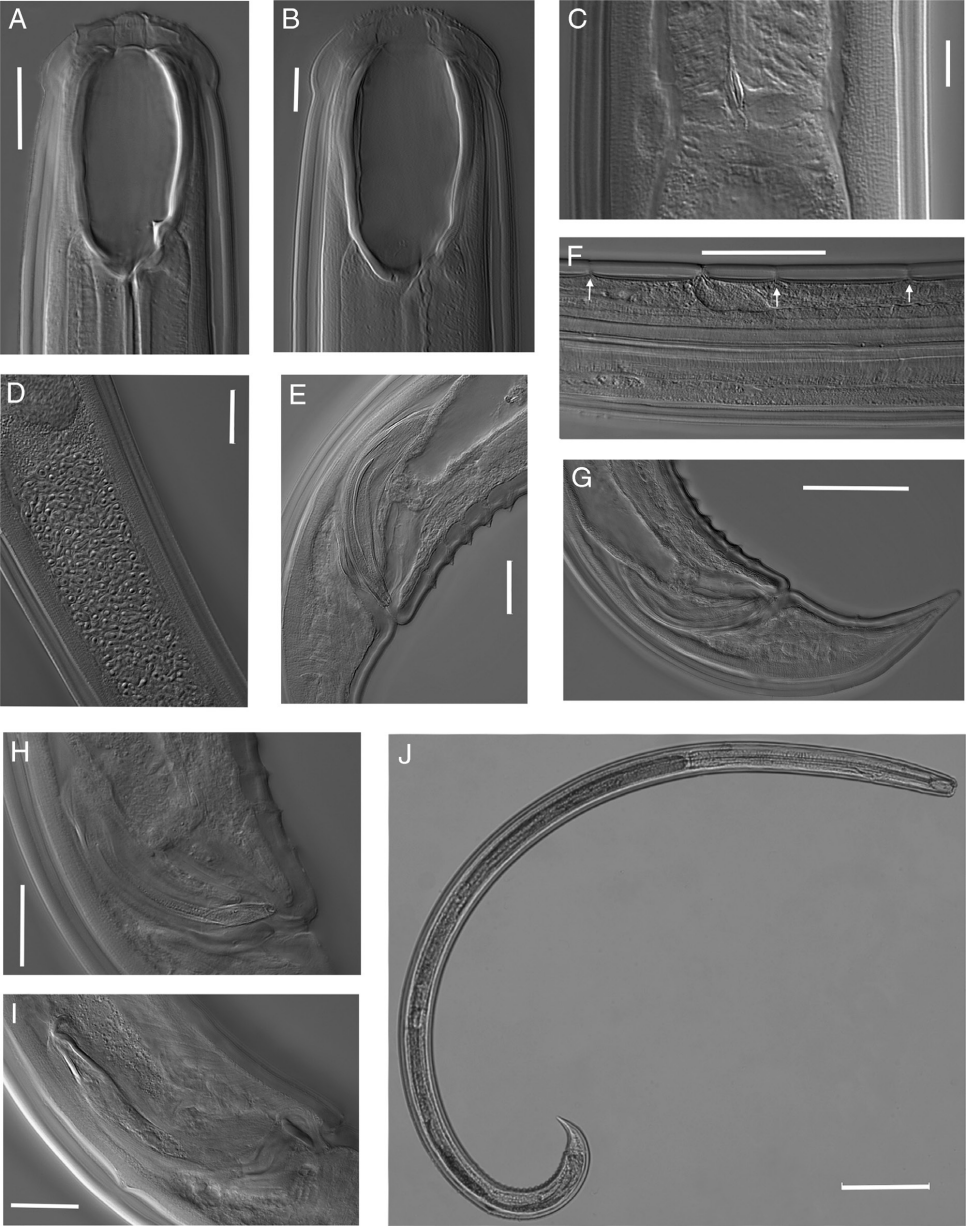
Light micrographs of *Parkellus hagiangensis* sp. nov. Male. A. Anterior end, median section, with the focus on the dorsal tooth. B. Anterior end, median section, with the focus on the longitudinal ridge. C. Pharyngo-intestinal junction. D. Sperm cells. E. Distal part of spicules. F. Excretory pore and ventromedian cuticular pores above and below of the excretory pore. G. Tail. H. Lateral guiding pieces. I. Proximal part of spicules. J. Entire body. Scale bar: J = 200 µm, F, G = 50 µm, A, D, E, H, I = 20 µm, B, C = 10 µm.

**Figure 3:**
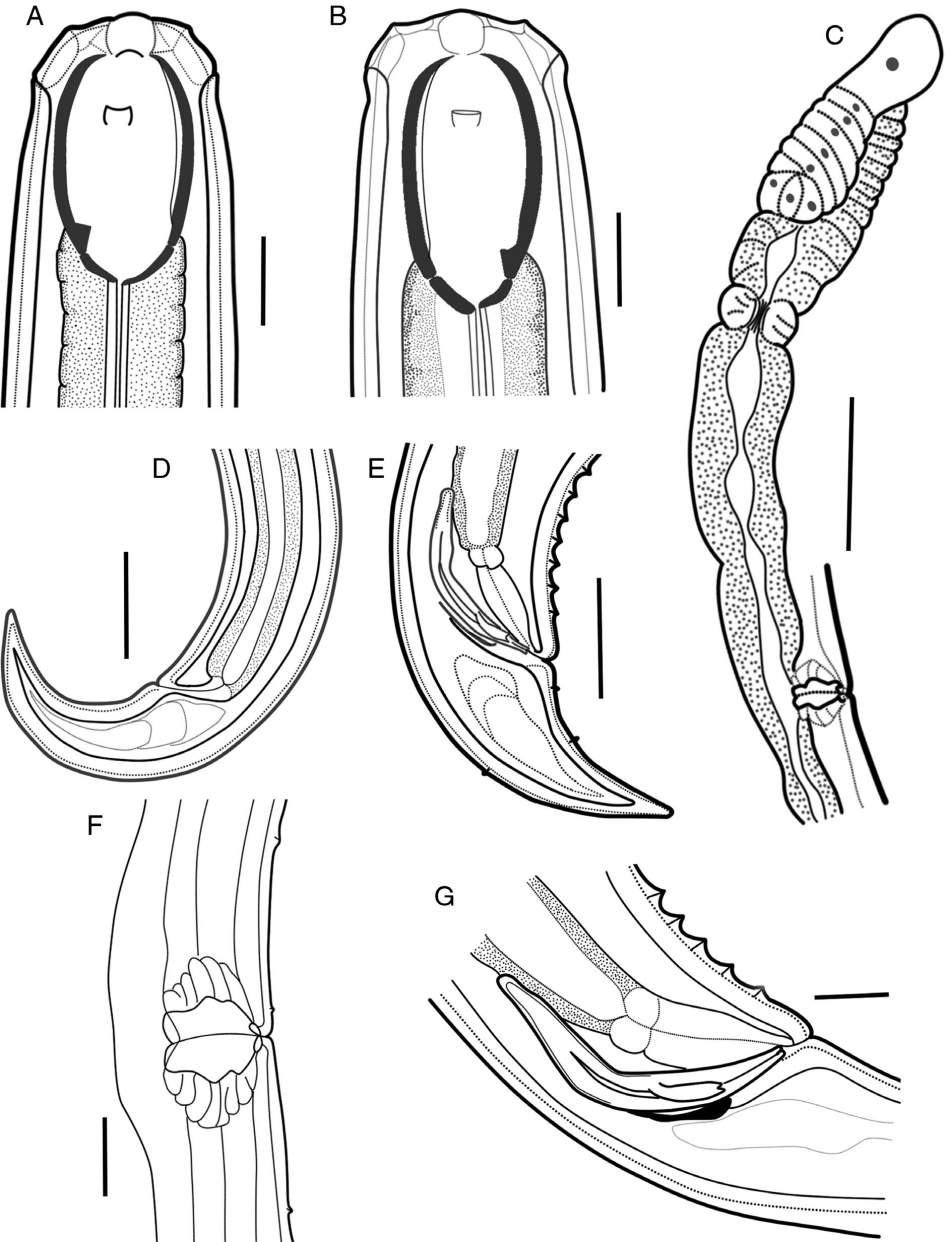
Line drawings of *Parkellus hagiangensis* sp. nov. A. Anterior end of female, median section. B. Anterior end of male, median section. C. Female anterior genital branch. D. Female tail. E. Male tail. F. Vulval region with advulval ventromedian papillary structures. G. Male copulatory apparatus. Scale bar: A, B, F, G = 20 µm, C–E = 50 µm.

*Parkellus hagiangensis*sp. nov.

(Figs. 1–3 and Table 1)

**Table 1.** Morphometrics of females and males of *Parkellus hagiangensis* sp. nov. from Ha Giang Province, Vietnam.

	Du Gia natural reserve		
Characters/ratios*	Holotype	Paratypes	
n	1 female	1 female	3 males
L (µm)	2,112	1,769	2,162–2,900
a	26.0	27.2	29.0–34.9
b	3.9	3.7	3.8–4.1
c	16.4	15.2	21.4–23.2
c′	2.8	2.7	1.7–2.1
V	69	70	
Lip region height (µm)	13.3	12.2	12.0–13.6
Lip region width (µm)	40.5	40.2	44.8–46.4
Buccal cavity length (µm)	54.4	50.8	55.4–60.2
Buccal cavity width (µm)	29.9	29.1	28.8–31.6
Position of tooth apex (%) from anterior end of buccal cavity	75	76	71–74
Nerve ring from anterior end (µm)	143	138	152–170
Excretory pore from anterior end (µm)	173	153	177–196
Pharynx length (µm)	539	473	566–713
Maximum body width (µm)	78.3	65.0	74.7–83.0
Anal body width (µm)	45.5	42.7	55.0–65.2
Rectum/cloaca length (µm)	38.7	33.4	41.0–51.4
Spicules length (µm)			82.3–97.3
Lateral guiding piece length (µm)			18.2–22.2
Tail length (µm)	128	116	95–136

**Notes:***L* = body length; *a* = body length/maximum body width; *b* = body length/pharyngeal length; *c* = body length/tail length; *c′* = tail length/body width at anus; *V* = (distance from anterior end to vulva/body length) x 100.

### Type material

Holotype female and two paratype males, in permanent mounts in glycerine, deposited in the nematode collection at the Department of Nematology, Institute of Ecology and Biological Resources, Vietnam Academy of Science and Technology. One paratype female and one paratype male deposited in the nematode collection of the Museum and Institute of Zoology, PAS, Warsaw, Poland.

### Type habitat and locality

Soil samples from pristine forest containing specimens originated from Du Gia natural conservation area at Minh Ngoc Commune, Bac Me District, Ha Giang Province (N 22°42′57″, E 105°11′45″, 350 m a.s.l.).

### Etymology

The name of the species refers to its geographical origin from Ha Giang Province in Vietnam.

### Description

Measurements see Table 1.

### Adult

Relaxed specimens arcuate, more curved ventrally at posterior end. Body tapering slightly anterior to base of pharynx but more sharply toward posterior end. Maximum body width at the level of vulval region. Cuticle smooth, 6.4 (5.3–7.5) μm thick at the base of pharynx. Sublateral, subdorsal, and subventral body pores distinct along the entire body excluding tail.

Lip region rounded, 3.4 (3.1–3.7) times as wide as high, slightly offset by a depression, its diameter almost the same as the adjacent body. Labial papillae small, conical, slightly protruding. Cephalic papillae comparatively more prominent, broadly rounded, slightly protruding beyond the body outline. Amphidial fovea cup-shaped, its aperture 5.4 (5.1–5.8) µm wide, located 20.8 (18.6–22.8) µm from the anterior body end. Buccal cavity spacious, oval-shaped, 1.8 (1.7–2.1) times as long as wide, with funnel-shaped base, its wall strongly sclerotized. Dorsal tooth medium-sized, located at the base of the vertical dorsal plate of the buccal cavity, below the beginning of the pharynx. Apex of dorsal tooth located 41.1 (38.1–44.6) µm or 71–76% of buccal cavity length from its anterior end, opposite to it lies a thin, subventral longitudinal ridge. Nerve ring encircling cylindrical, muscular pharynx at about 26.5 (23.8–29.2%) of its length. Excretory pore distinct, males have there are three well developed ventral pores, first one at 40.7–46.6 µm above and the next two at 24.7–29.7 and 74.3–82.6 µm behind the excretory pore. Pharyngo-intestinal junction non-tuberculate. The intestine content nematodes, diatom shells, earthworm setae and detritus are visible. Rectum wide, arcuate, 0.8 times the anal body diameter long. Tail conical, ventrally bent, regularly tapering, with rounded tip. Hyaline part well developed, 17.0 (10.9–23.8)  µm long. Caudal glands and terminal opening absent.

#### Female:

Genital system didelphic-amphidelphic, the sexual branches almost equally developed, anterior 225–238 µm long or 11–13% of body length, posterior 140–187 µm long or 8–9% of body length. Ovaries on alternate sides of the intestine, well developed, reflexed with numerous oocytes, not reaching the uterus-oviduct junction, oviduct takes the form of a slender tube, which then expands to form a well-developed *pars dilatata* with distinct lumen, it is connected with the uterus by a muscular sphincter, uterus, relatively long, contains numerous sperm cells mainly in its distal part. Vagina extending inwards for 27.8–28.3 µm or 35–43% of body diameter, *pars proximalis vaginae* longer than wide, pitcher-shaped, surrounded by strong circular muscles, *pars refringens vaginae* with a very faint, small (6.7 x 4.7 µm), teardrop-shaped pieces, *pars distalis vaginae* short, vulva a transverse slit. Small, ventromedian vulval papillae, one or two on each side of the vulva are visible. Female tail with four pairs of sublateral caudal pores.

#### Male:

Genital system diorchic, testes opposed. Sperm cells elongate spindle shaped. Spicules total length along arc 1.2 times that at chord, 1.4–1.5 times longer than body diameter at cloacal aperture, relatively thin, 9.1–9.4 times longer than wide ventrally curved, curvature 114–119° median piece marked between hump and lateral guiding pieces, occupying 39.9–41.3% of spicule total length. Dorsal contour arcuate, ventral side with moderately expressed hump and hollow, the former located at 27.3–28.7% of spicule total length from its anterior end, head oval-shape, offset by a shallow depression, proximal part of spicules conical, posterior end bifurcate, 4.4–5.0 µm broad. Lateral guiding pieces with arched edges and bifurcate terminus, this furcation is symmetrical and poorly marked. Gubernaculum well developed, 31.9–34.7** **µm long. Ventromedian supplements 19–23 in number, conical and regularly arranged, occupies 12.9–13.3% of the body length, the anterior most situated at 307–326 µm from cloacal aperture, distance between the first and last supplement 287–307 µm. Above the series of supplements, 1–2 cuticular pores are visible. Male tail with two pairs of sublateral, two pairs of subdorsal caudal pores and two pairs of small subventral papillae.

### Diagnosis

*Parkellus hagiangensis* sp. nov. is characterized by its large sized body (1,769–2,900 µm); males having the ventromedian cuticular pores above and below of the excretory pore; position of amphidial aperture (18.6–22.8 µm from the anterior body end); 50.8–60.2 µm long buccal cavity; dorsal tooth apex at 38.1–44.6 µm from anterior margin of buccal cavity or 71–76% of buccal cavity length, located at the base of the vertical dorsal plate of the buccal cavity, below the beginning of the pharynx; *pars refringens vaginae* with very faint and small (6.7 x 4.7 µm) teardrop-shaped pieces; short *pars distalis vaginae*; small ventromedian vulval papillae and 82.3–97.3 µm long spicules with rounded head and conical proximal part. *P. hagiangensis* differs from all so far known *Parkellus* species by having more posterior dorsal tooth apex from anterior margin of buccal cavity.

In general appearance *Parkellus hagiangensis* sp. nov. is similar to *P. parkus* (Jairajpuri et al., 2001), *P. zschokkei* (Menzel, 1913), and *P. tuyenquangensis* sp. nov. From *P. parkus,* it is distinguished by having lower position of dorsal tooth apex (71–76% vs 61–65%), longer female tail (116–128 vs 85–90 µm) and males having the ventromedian cuticular pores above and below of the excretory pore. From *P. zschokkei* it differs by lower position of dorsal tooth apex (71–76% vs 47–60%), lower position of amphidial aperture (18.6–22.8 vs 12.0–16.0 µm) from anterior body end, males having the ventromedian cuticular pores above and below of the excretory pore and spicule curvature (114–119° vs 104–109°). From *P. tuyenquangensis* sp. nov. it is distinguished by having lower position of dorsal tooth apex (71.0–76.0% vs 60.0–70.0%), appearance of very faint and small *pars refringens vaginae* pieces vs strong sclerotized and medium size, structure of *pars distalis vaginae* (not thickened vs thickened at the junction with *pars refringens vaginae*), presence of small vulval papillae (vs absence), structure of proximal part of spicule (conical vs cylindrical).

### Molecular characterization

Nearly full length (1,600 bp), identical 18S rDNA sequences were acquired from two individuals belonging to *P. hagiangensis* sp. nov. (MT799665) while identical, 1 kb long, 28S rDNA sequences were obtained from three individuals (MT799670). The results of the phylogenetic analyses using these molecular data are presented in Figures 9 and 10.

**Figure 4:**
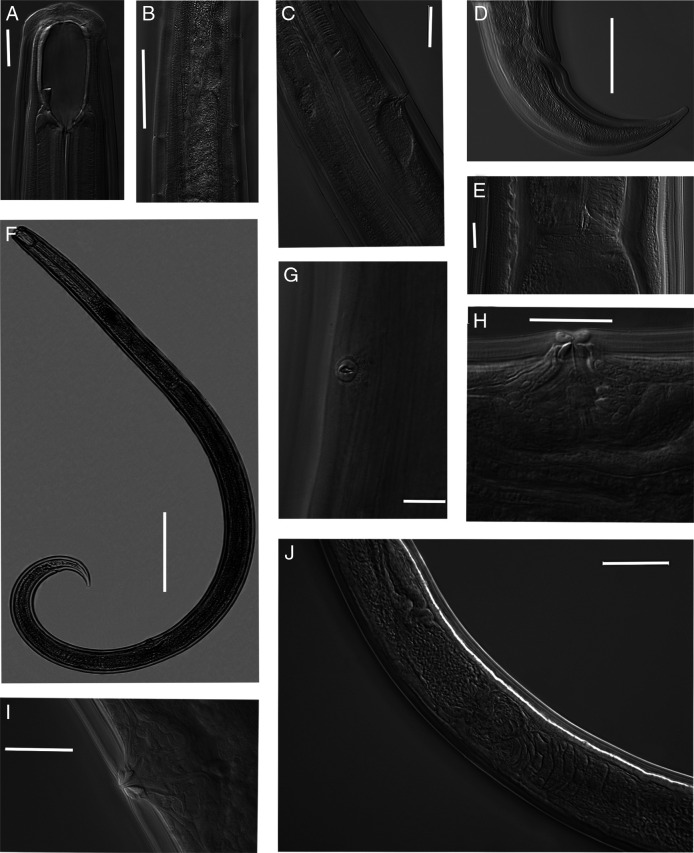
Light micrographs of *Parkellus tuyenquangensis* sp. nov. Female. A. Anterior end, median section, with the focus on the dorsal tooth. B. Body pores. C. Anterior genital branch. D. Entire body. E. Excretory pore. F. Tail. G. Pharyngo-intestinal junction. H, J. Vulval region. Scale bar: D = 200 µm, B, C = 50 µm, F = 45 µm, A, E, H–J = 20 µm, G = 10 µm.

**Figure 5:**
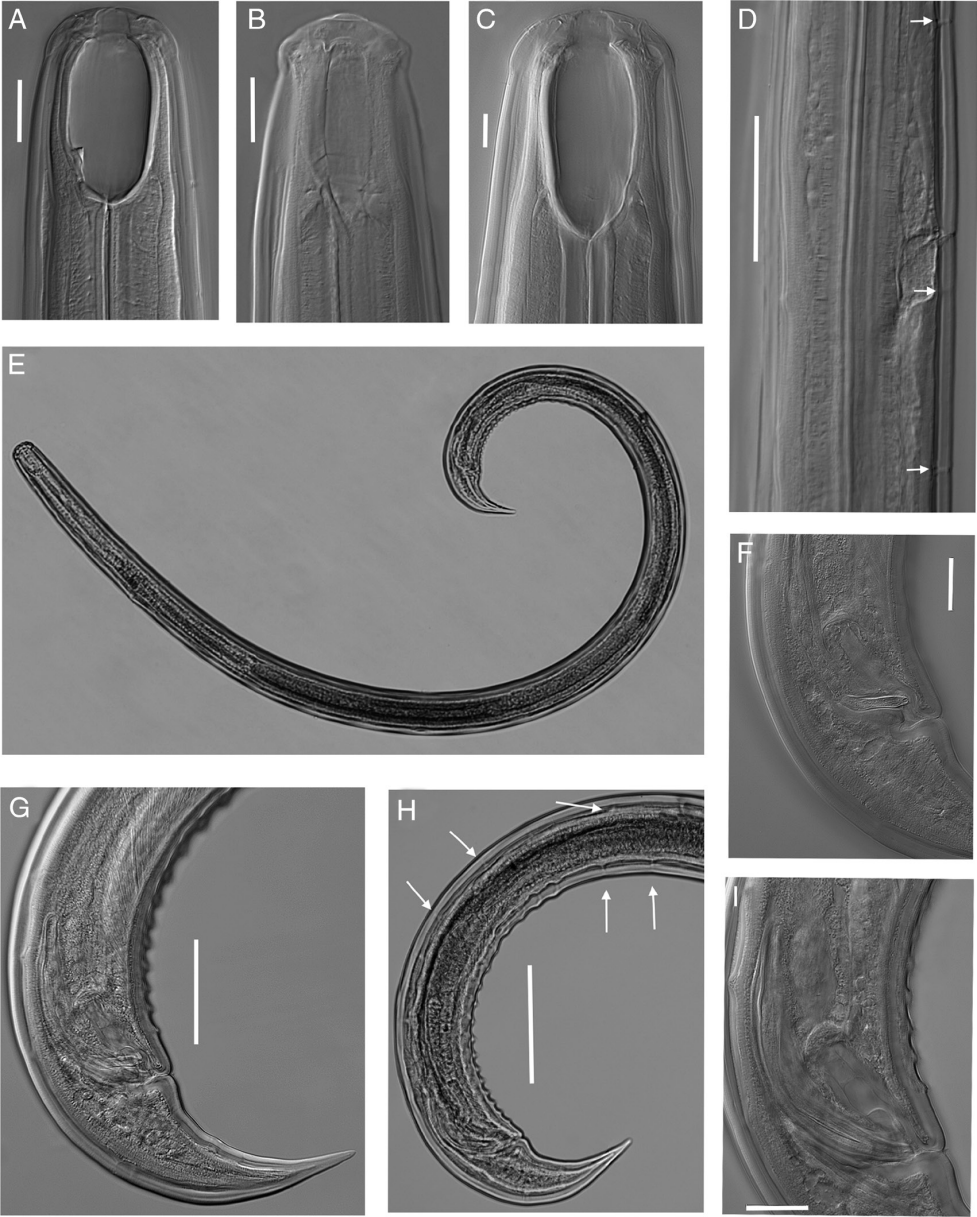
Light micrographs of *Parkellus tuyenquangensis* sp. nov. Male. A. Anterior end, median section, with the focus on the dorsal tooth. B. Anterior end, surface view. C. Anterior end, median section, with the focus on the longitudinal ridge. D. Excretory pore and ventromedian cuticular pores above and below of the excretory pores. E. Entire body. F. Lateral guiding pieces. G. Tail. H. Posterior end. I. Proximal part of spicules. Scale bar: E = 200 µm, H = 100 µm, D, G = 45 µm, A, B, F, I = 20 µm, C = 10 µm.

**Figure 6:**
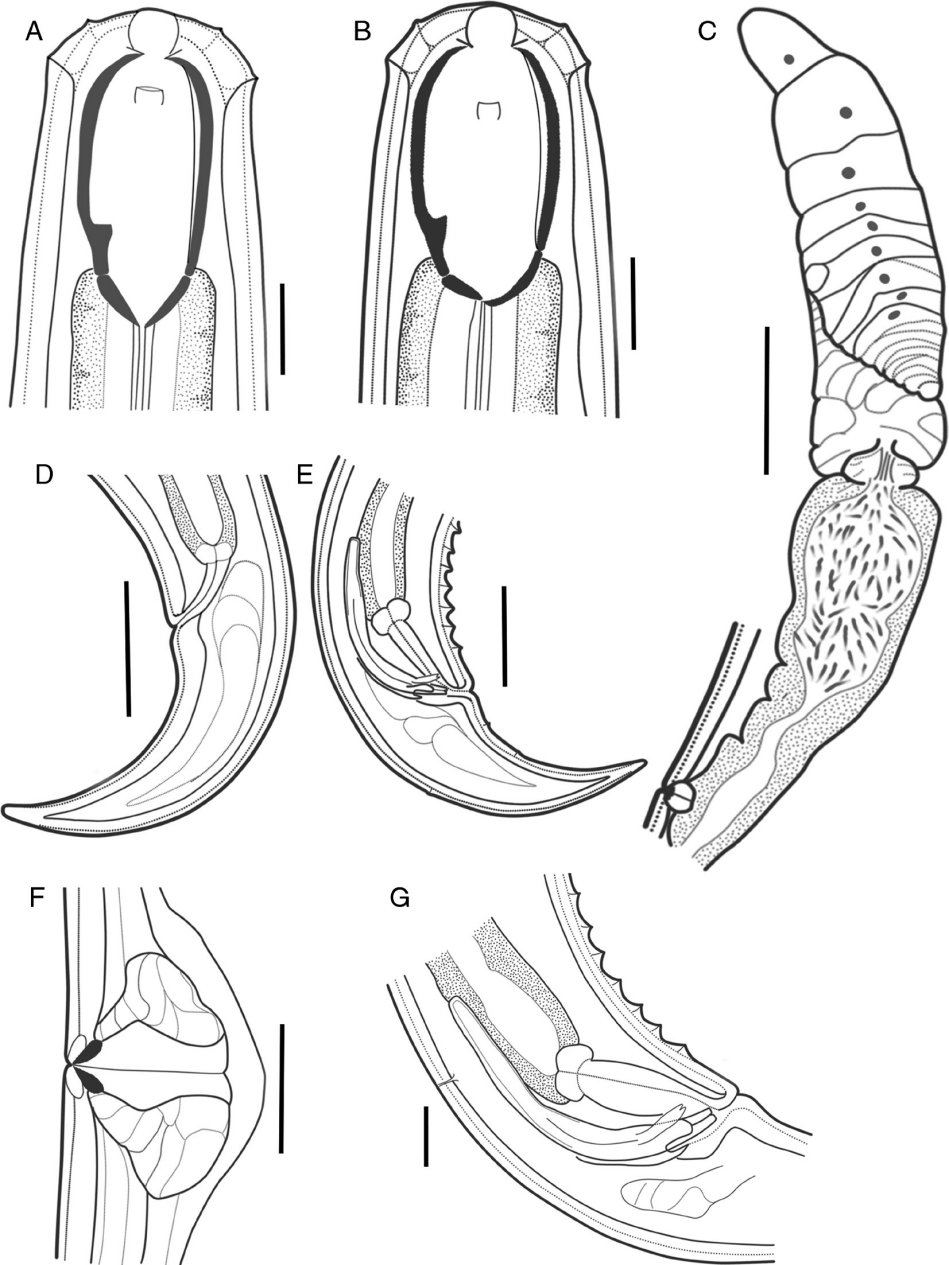
Line drawings of *Parkellus tuyenquangensis* sp. nov. A. Anterior end of female, median section. B. Anterior end of male, median section. C. Female anterior genital branch. D. Female tail. E. Male tail. F. Vulval region with advulval ventromedian papillary structures. G. Male copulatory apparatus. Scale bar: A, B, F, G = 20 µm, C–E = 50 µm.

*Parkellus tuyenquangensis* sp. nov.

(Figs. 4–6 and Table 2)

**Table 2.** Morphometrics of females and males of *Parkellus tuyenquangensis* sp. nov. from Tuyen Quang Province, Vietnam.

	Cham Chu natural reserve				
Character	Holotype	Paratypes		Na Hang natural reserve	
n	female	3 females	3 males	11 females	12 males
L (µm)	2,205	2,185–2,487	2,235–2,648	2,995 ± 221.4 (2,726–3,363)	2,753 ± 152.8 (2,545–3,067)
a	29.8	25.5–31.8	28.7–31.7	30.5 ± 2.0 (28.0–30.0)	32.8 ± 2.0 (28.4–34.6)
b	3.6	3.8–4.0	3.8–4.0	4.4 ± 0.2 (4.1–4.7)	4.2 ± 0.1 (4.0–4.5)
c	17.2	18.2–19.8	17.3–26.3	19.7 ± 1.4 (18.1–22.4)	21.8 ± 1.1 (19.7–23.5)
c′	2.6	2.5–2.7	1.7–2.1	2.8 ± 0.2 (2.3–3.0)	2.1 ± 0.1 (1.9–2.3)
V	68.0	69.0		68.7 ± 2.9 (67.0–73.0)	
Lip region height (µm)	12.6	11.1–14.6	12.3–13.9	10.6 ± 0.8 (8.8–11.4)	11.3 ± 1.2 (9.7–13.2)
Lip region width (µm)	43.1	40.9–47.4	41.2–43.8	45.4 ± 2.8 (41.4–50.2)	44.8 ± 1.4 (43.0–46.6)
Buccal cavity length (µm)	57.7	52.5–60.4	53.9–58.7	56.6 ± 1.7 (54.6–60.7)	55.4 ± 1.6 (53.0–58.0)
Buccal cavity width (µm)	28.2	28.0–29.3	27.7–28.5	30.1 ± 0.7 (29.0–30.8)	29.0 ± 1.0 (27.3–30.8)
Position of tooth apex (%) from anterior end of buccal cavity	64.0	60.0–62.0	60.0–67.0	66.4 ± 1.6 (63.0–68.3)	66.9 ± 1.8 (64.0–70.0)
Nerve ring from anterior end (µm)	149	140–163	155–163	172 ± 11.5 (151–191)	170 ± 8.3 (158–180)
Excretory pore from anterior end (µm)	185	175–195	180–194	204 ± 11.0 (189–223)	199 ± 9.8 (185–216)
Pharynx length (µm)	606	543–635	569–655	686 ± 35.0 (636–750)	657 ± 26.6 (613–704)
Maximum body width (µm)	73.9	78.1–83.4	78.0–83.6	98.6 ± 9.9 (81.0–110.0)	86.1 ± 5.9 (76.5–96.8)
Anal body width (µm)	48.3	47.6–50.8	59.4–62.7	55.5 ± 2.5 (49.5–58.5)	61.2 ± 2.0 (58.5–65.0)
Rectum/cloaca length (µm)	39.9	40.1–42.2	49.0–60.0	46.0 ± 4.2 (38.3–51.8)	54,7± 3,9 (50.1–64.2)
Spicules length (µm)			97.0–104.8		103.7 ± 5.8 (95.0–110.0)
Lateral guiding piece length (µm)			12.6–17.8		16.3 ± 0.9 (15.0–17.6)
Tail length (µm)	128	120–127	101–129	152 ± 13.6 (122–169)	126.4 ± 7.6 (110–133)

**Notes:***L* = body length; *a* = body length/maximum body width; *b* = body length/pharyngeal length; *c* = body length/tail length; *c′* = tail length/body width at anus; *V* = (distance from anterior end to vulva/body length) x 100.

### Type material

Holotype female, three paratype females, and three paratype males from Cham Chu and Na Hang population, in permanent mounts in glycerin, deposited in the nematode collection at the Department of Nematology, Institute of Ecology and Biological Resources, Vietnam Academy of Science and Technology. Nine paratype females and nine paratype males from Cham Chu and Na Hang population deposited in the nematode collection of the Museum and Institute of Zoology, PAS, Warsaw, Poland. Two paratype females and three paratype males from Na Hang population deposited in State Museum of Natural History NASU, Lviv, Ukraine.

### Type habitat and locality

Cham Chu population: soil samples from pristine forest containing specimens originated from Cham Chu natural conservation area at Yen Thuan Commune, Ham Yen District, Tuyen Quang Province (N 22°17′55″,E 104°59′42″, 820 m a.s.l.).

Na Hang population: soil samples from pristine forest containing specimens originated from Na Hang natural conservation area at Na Hang District, Tuyen Quang Province (N 22°20′53″,E 105°25′49″, ca 320 m a.s.l.).

### Etymology

The name of the species refers to its geographical origin from Tuyen Quang Province in Vietnam.

### Description

Measurements, see Table 2.

### Adult

Relaxed specimens arcuate, more curved ventrally at posterior end. Body tapering slightly anterior to base of the pharynx but more sharply toward the posterior end. Maximum body width at the level of vulva. Cuticle smooth, 7.1 (6.2–8.5) μm thick at the base of the pharynx. Sublateral, subdorsal, and subventral body pores visible along the entire body excluding tail.

Lip region rounded, 3.4 (3.0–3.7) times as wide as high, slightly offset by a depression, its diameter slightly wider than an outline of the adjacent body. Labial papillae small, conical, slightly protruding. Cephalic papillae bigger, broadly rounded, slightly protruding beyond the body outline. Amphidial fovea cup-shaped, its aperture 5.2 (4.2–6.4)  µm wide, located 19.5 (17.7–22.0)  µm from the anterior body end. Buccal cavity spacious, oval-shaped, 2.0 (1.9–2.1) times as long as wide, with funnel-shaped base. Dorsal tooth small, located at the vertical dorsal plate of the buccal cavity, above the beginning of the pharynx. Apex of dorsal tooth located 35.7 (31.6–37.7) µm from anterior margin of the buccal cavity or 60–70% of the buccal cavity length from its anterior end, opposite to it lies a thin, subventral, longitudinal ridge. Nerve ring encircling cylindrical, muscular pharynx at about 25.6 (22.0–28.6%) of its length. Excretory pore, ampulla and renette cells well visible. Males with three or four very well-developed ventral pores near the excretory pore, first one located at 75.4 (71.9–83.8) µm and the second one (in three specimens only) at 22.0–52.4 µm above the excretory pore, the next two at 39.0 (15.6–47.4) and 83.4 (72.0–89.5) µm behind the excretory pore. Pharyngo-intestinal junction non-tuberculate. In the intestine content visible nematodes, diatom shells, earthworm setae and detritus. Rectum wide and arcuate, 0.8–0.9 times the anal body diameter long. Tail conical, ventrally bent, regularly tapering, with rounded tip. Hyaline part well developed, 16.8 (14.9–21.7) µm long. Caudal glands and terminal opening absent.

#### Female:

Genital system didelphic-amphidelphic, the sexual branches almost equally developed, anterior 364 (291–430)  µm long or 12.9 (11.2–15.0%) of body length, posterior 322 (223–384)  µm long or 11.4 (8.2–13.4%) of body length. Ovaries on alternate sides of intestine, well developed, reflexed with numerous oocytes, not reaching the uterus-oviduct junction, oviduct takes the form of a slender tube, which then expands to form a well-developed *pars dilatata* with distinct lumen, it is connected with the uterus by a muscular sphincter, inner part of this sphincter heavily sclerotized, uterus, relatively long, contains numerous sperm cells mainly in its distal part, sometimes spermatozoids are also visible in sphincter and proximal part of *pars dilatata*. Vagina extending inwards for 29.1 (26.2–33.4) µm or 29.8 (24.8–35.7%) of body diameter, *pars proximalis vaginae* longer than wide, pitcher-shaped, surrounded by strong circular muscles, *pars refringens vaginae* medium size (9.0–12.9 × 4.8–8.0 µm), very strongly sclerotized, teardrop-shaped pieces, *pars distalis vaginae* very short, thickened at the junction with *pars refringens vaginae*, vulva transverse slit, sometimes the vulval lips extend beyond the body contour. No papillary structures before and behind the vulva, sometimes only single cuticular pores are visible, one on each side of vulva. Female tail with four pairs sublateral caudal pores.

#### Male:

Genital system diorchic, with opposed testes. Sperm cells elongate spindle-shaped. Ejaculatory glands in tandem, generally distinct. Spicules total length along arc 1.2–1.3 times that at chord, 1.5 (1.4–1.9) times longer than body diameter at cloacal aperture, relatively thin, 9.2 (7.8–10.1) times longer than wide, ventrally curved, curvature 115 (112–119º), median piece marked between hump and lateral guiding pieces, occupying 36.7% (31.0–40.5%) of spicule total length. Dorsal contour arcuate, ventral side with moderately expressed hump and hollow, the former located at 24.4% (18.4–29.6%) of spicule total length from its anterior end, head oval-shape, proximal part of spicule almost cylindrical, posterior end bifurcate, 5.4 (4.6–6.0)  µm broad. Lateral guiding pieces with arched edges and bifurcate terminus, this furcation is symmetrical and poorly marked. Gubernaculum well developed, 33.9 (30.1–40.6) µm long. Ventromedian supplements 16–20 in number, conical, regularly arranged, occupies 12.9% (12.1–14.8%) of body length, the anterior most situated at 19.1 (15.7–29.0)  µm from cloacal aperture, distance between the first and last supplement 334 (240–408) µm. Above the supplements visible two cuticular pores. Male tail with two pairs of sublateral and two pairs of subdorsal caudal pores and two pairs of small subventral papillae.

### Diagnosis

*Parkellus tuyenquangensis* sp. nov. is characterized by its large sized body (2,185–3,363  µm); males having the ventromedian cuticular pores above and below of the excretory pore; 52.5–60.7 µm long buccal cavity; dorsal tooth apex at 31.6–37.7 µm from anterior margin of buccal cavity or 60–70% of buccal cavity length); *pars refringens vaginae* with a very strongly sclerotized, medium size (9.0–12.9 × 4.8–8.0 µm) teardrop-shaped pieces; *pars distalis vaginae* very short and thickened at the junction with *pars refringens vaginae*; and spicules 95–110 µm long with rounded head and cylindrical proximal part and lateral guiding pieces 13–18 µm long.

In general appearance *Parkellus tuyenquangensis* sp. nov. is similar to *P. parkus* (Jairajpuri et al., 2001), *P. zschokkei* (Menzel, 1913), and *Parkellus hagiangensis* sp. nov. From *P. parkus* it is distinguished by males having the ventromedian cuticular pores above and below of the excretory pore (vs males without this kind of pores), longer spicules (95–110 vs 82–92 µm), length and shape of lateral guiding pieces (13–18 vs 22–25 µm), distal part barrel-shaped vs cylindrical) and longer female tail (120–169 vs 85–90 µm). From *P. zschokkei* it differs by the lower position of the dorsal tooth apex (60–70% vs 47–60%), lower position of amphidial aperture (17.7–22.0 vs 12.0–16.0 µm) from anterior body end lack of ventromedian vulval papillae, males having the ventromedian cuticular pores above and below of the excretory pore (vs males without this kind of pores) spicule curvature (112–119° vs 104–109°). The differences between *Parkellus tuyenquangensis* sp. nov. and *Parkellus hagiangensis* sp. nov. were described above in the diagnosis in the description of *Parkellus hagiangensis* sp. nov.

### Molecular characterization

For the *P. tuyenquangensis* sp. nov. we were able to obtain only the second part of the 18S rDNA gene (nearly 800 bp; MT799666) from one of the two molecularly-analyzed nematode individuals. Identical, 1 kb long, 28S rDNA sequences were successfully obtained from both of these nematodes (MT799671). The results of the phylogenetic analyses using these molecular data are presented in Figures 9 and 10.

**Figure 7:**
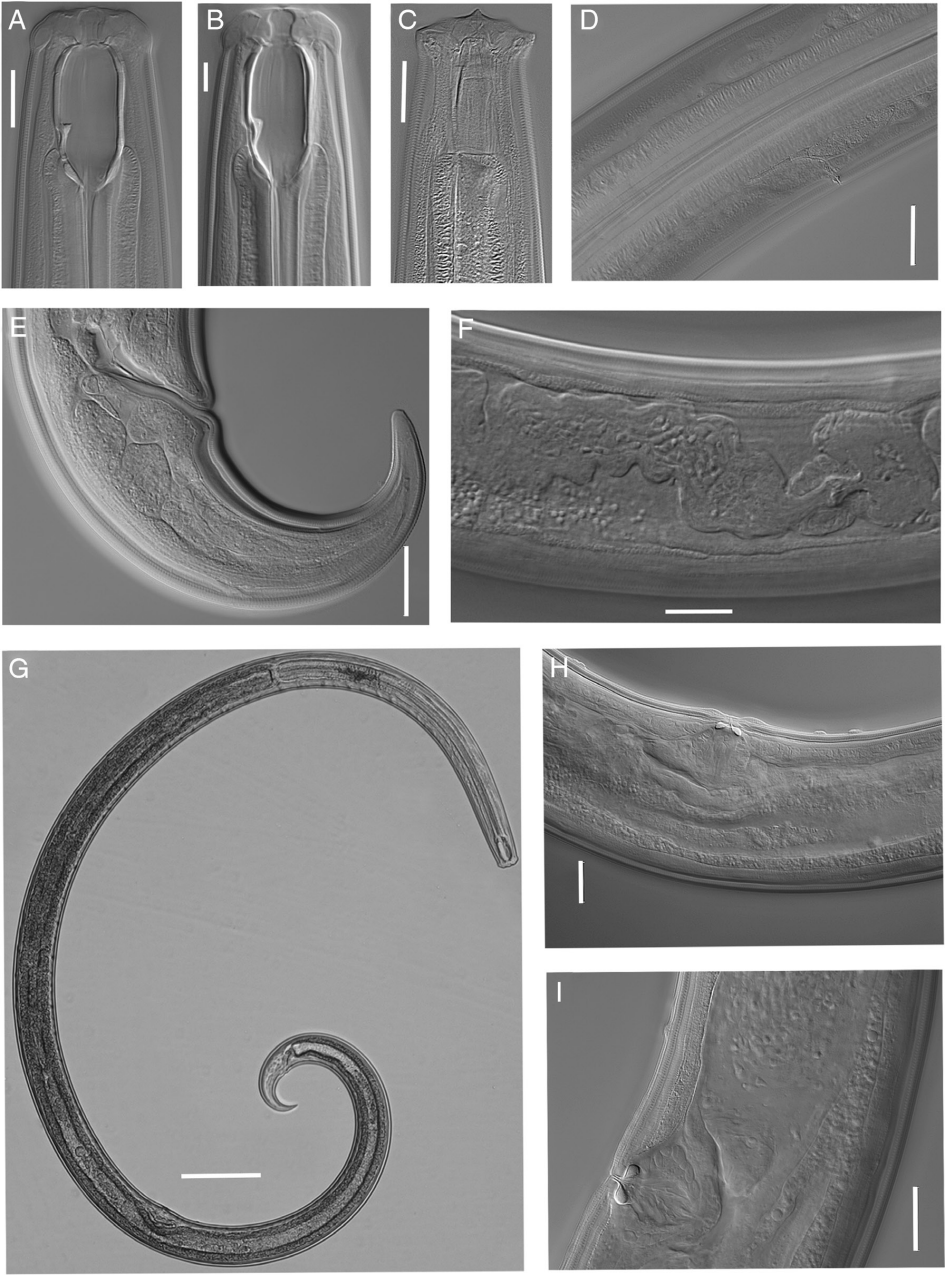
Light micrographs of *Parkellus zschokkei*. Female. A. Anterior end, median section, with the focus on the dorsal tooth. B. Anterior end, median section, with the focus on the longitudinal ridge. C. Anterior end, surface view. D. Excretory pore. E. Tail. F. Oviduct-uterus sphincter. G. Entire body. H. Vulval region with advulval ventromedian vulval papillae. I. Vulval region. Scale bar: G = 200 µm, H = 50 µm, A, C–F, I = 20 µm, B = 10 µm.

**Figure 8:**
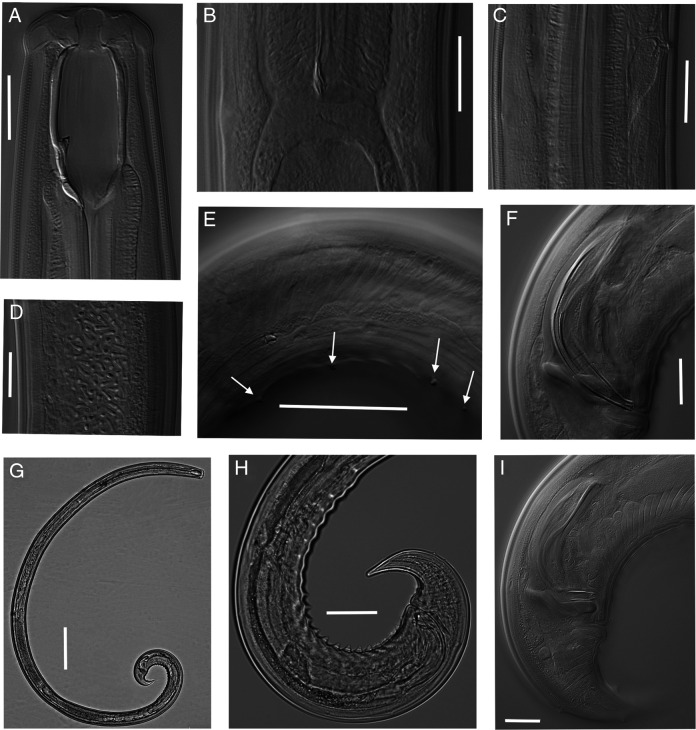
Light micrographs of *Parkellus zschokkei*. Male. A. Anterior end, median section, with the focus on the dorsal tooth. B. Pharyngo-intestinal junction. C. Excretory pore. D. Sperm cells. E. Subventral papillae lying parallel to the supplements. F. Spicules. G. Entire body. H. Posterior end. I. Lateral guiding pieces. Scale bar: G = 200 µm, E, H = 50 µm, A–D, F, I = 20 µm.

*Parkellus zschokkei* (Menzel, 1913)

(Figs. 7 and 8 and Table 3)

**Table 3.** Morphometrics of females and males of *Parkellus zschokkei* (Menzel, 1913) from Ukraine.

Characters/ratios*	Uzhhorod	
n	13 females	11 males
L (µm)	2900 ± 175.3 (2,569–3,174)	2748 ± 152.5 (2,454–3,012)
a	33.0 ± 2.4 (29.3–38.8)	36.6 ± 1.4 (34.1–39.7)
b	4.5 ± 0.2 (4.3–4.8)	4.3 ± 0.1 (4.0–4.5)
c	21.4 ± 1.7 (17.4–23.1)	23.4 ± 2.1 (21.0–27.8)
c′	2.5 ± 0.2 (2.2–2.9)	1.8 ± 0.2 (1.5–2.0)
V	68.0 ± 1.3 (65.0–70.3)	
Lip region height (µm)	12.8 ± 1.1 (10.1–13.9)	12.4 ± 1.0 (10.6–13.8)
Lip region width (µm)	42.7 ± 2.1 (38.7–46.8)	42.4 ± 1.9 (39.4–44.7)
Buccal cavity length (µm)	50.8 ± 1.6 (48.3–53.7)	51.2 ± 1.8 (47.1–54.5)
Buccal cavity width (µm)	24.9 ± 1.0 (23.3–26.6)	25.0 ± 0.5 (23.8–25.5)
Position of tooth apex (%) from anterior end of buccal cavity	54.5 ± 3.1 (47.4–59.5)	55.8 ± 2.1 (51.7–58.4)
Nerve ring from anterior end (µm)	189 ± 10.0 (169–202)	188 ± 8.9 (171–200)
Excretory pore from anterior end (µm)	218 ± 12.0 (197–241)	218 ± 7.3 (202–229)
Pharynx length (µm)	642 ± 35.2 (579–705)	641 ± 21.1 (606–667)
Maximum body width (µm)	88.0 ± 5.3 (75.3–93.2)	74.9 ± 4.8 (65.7–82.1)
Anal body width (µm)	53.4 ± 2.6 (50.8–60.4)	66.4 ± 5.1 (60.1–75.8)
Rectum/cloaca length (µm)	45.7 ± 2.5 (42.8–50.2)	54.5 ± 2.6 (50.1–59.0)
Spicules length (µm)		106 ± 2.9 (102–111)
Lateral guiding piece length (µm)		21.5 ± 1.3 (20.3–24.5)
Tail length (µm)	136 ± 8.6 (120–151)	118 ± 10.8 (94–134)

### Material examined

Specimens of *Parkellus zschokkei* from three Ukrainian populations: (I) Uzhhorod, Zakarpattia region, Ukraine, oak forest (N 48°38′39.54″; E 22°18′11.78″, 191 m a.s.l.); (II) Uzhhorod, Zakarpattia region, Ukraine, oak forest (N 48°38′02.67″; E 22°20′40.13″, 154 m a.s.l.); (III) Uzhhorod, Zakarpattia region, Ukraine, oak forest N 48°35′47.88″; E 22°22′41.51″, 170 m a.s.l.). In total, 13 females and 11 males (population I) fixed with 4% TAF, mounted in glycerine, on permanent microscope slides and 16 females and 6 males (population I, II, III) fixed with DESS mixture and submitted for molecular study (see Material and methods).

### Description

Measurements see Table 3.

### Adult

Relaxed specimens arcuate, more curved ventrally at posterior end. Body tapering slightly anterior to base of pharynx but more sharply toward posterior end. Maximum body width at the level of vulva. Cuticle smooth, 4.3 (3.1–5.1) μm thick at the base of pharynx, subcuticle with faint transverse striae. Sublateral, subdorsal, and subventral body pores distinct along the entire body excluding tail. Tail with four pairs of sublateral caudal pores.

Lip region rounded, 3.4 (3.0–4.0) times as wide as high, offset by a depression, wider than outline of the adjacent body. Labial and cephalic papillae conical, protruding beyond of the body contour, cephalic papillae comparatively more prominent. Amphidial fovea cup-shaped, its aperture relatively large, 7.3 (6.4–9.5) µm wide, located 13.5 (12.0–16.0) µm from the anterior body end. Buccal cavity spacious, oval-shape, 2.0 (1.9–2.2) times as long as wide, with funnel-shaped base. Dorsal tooth small, at the vertical dorsal plate of the buccal cavity, above the beginning of the pharynx. Apex of dorsal tooth located 28.1 (24.7–30.3) µm from anterior margin of buccal cavity or 47–60% its length from anterior end, opposite to it lies a thin, subventral longitudinal ridge. Nerve ring encircling cylindrical, muscular pharynx at about 29.4% (27.7–31.5%) of its length. Excretory pore and the ampulla well marked. Pharyngo-intestinal junction non-tuberculate. In the intestine content visible nematodes, diatom shells, earthworm setae, and detritus. Rectum wide and arcuate, 0.8 (0.7–1.0) times the anal body diameter long. Tail conical, ventrally bent, regularly tapering, with rounded tip. Hyaline part well developed, 12.9 (9.4–18.6) µm long. Caudal glands and the terminal opening absent.

#### Female:

Genital system didelphic-amphidelphic, the sexual branches almost equally developed, anterior 348 (256–423) µm long or 12.1% (9.3–14.6%) of body length, posterior 349 (297–423) µm long or 12.1% (10.5–16.3%) of body length. Ovaries on alternate sides of intestine, reflexed and well developed with numerous oocytes, not reaching the uterus-oviduct junction, oviduct takes the form of a slender tube, which then expands to form a well-developed *pars dilatata* with a distinct lumen, it is connected with the uterus by a muscular sphincter having a heavily sclerotized inner part, uterus, relatively long, contains numerous spermatozoids mainly in its distal part, sometimes sperm cells are also visible in sphincter and proximal part of *pars dilatata*. Vagina extending inwards for 37.4 (34.3–41.0) µm or 43.2% (37.3–54.4%) of the body diameter, *pars proximalis vaginae* longer than wide, pitcher-shaped, surrounded by strong circular muscles, *pars refringens vaginae* with strong sclerotized, teardrop-shaped pieces, 13.8 (11.2–15.6) × 4.8 (4.1–6.0) µm, *pars distalis vaginae* very short, thickened at the junction with *pars refringens vaginae*, Vulva as a transverse slit, sometimes the vulval lips extend beyond the body contour. Small, ventromedian vulval papillae, one to three on each side of vulva are visible.

#### Male:

Genital system diorchic, with opposed testes. Sperm cells elongate spindle shaped. Ejaculatory glands in tandem, generally distinct. Spicules total length along arc 1.3–1.4 times that at chord, 1.6 (1.4–1.8) times longer than body diameter at cloacal aperture; relatively thin, 8.4 (8.1–8.6) times longer than wide, ventrally curved, curvature 105.6°(104–109.5°), median piece marked between hump and lateral guiding pieces, occupying 26.3% (22.0–30.1%) of spicule total length. Dorsal contour arcuate, ventral side with moderately expressed hump and hollow, the former located at 30.2% (26.4–32.9%) of spicule total length from its anterior end, head narrow, on a dorsal side offset by a shallow depression, proximal part of spicules almost cylindrical, posterior end bifurcate, 4.9 (4.2–5.3)  µm broad. Lateral guiding pieces with arched edges and a bifurcate terminus, this furcation is symmetrical and well-marked. Gubernaculum well developed, 41.6 (36.2–46.3)  µm long. Ventromedian supplements 21–24 in number, conical, regularly arranged, occupies 13.1% (11.8–13.9%) of body length. Above the supplements two cuticular pores are visible, the posterior most situated at 15.6 (13.8–16.6) µm from cloacal aperture, distance between the first and last supplement 360 (317–399) µm. Parallel to the supplements, on both sides of them, five to six subventral papillae of varying sizes are visible, largest one lies at the level of the anus. Almost at half of the tail length, on its ventral side a large medioventral caudal papilla is situated, below it lies one pair of subventral and two pairs of subdorsal papillae.

### Molecular characterization

**Figure 9:**
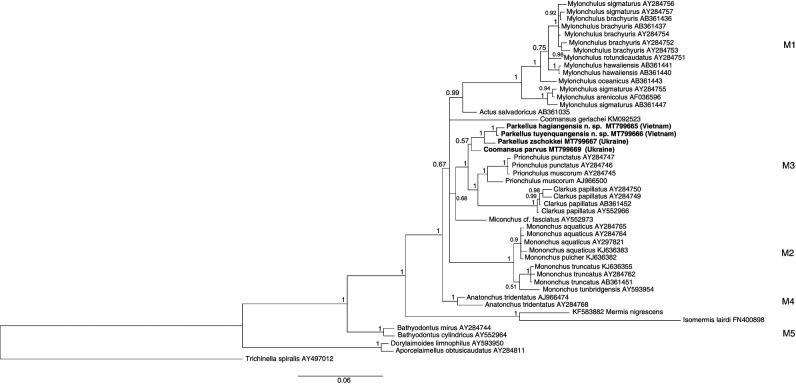
18S rDNA-based Bayesian phylogeny of the Mononchida. The new *Parkellus* species and the additionally studied *P. zschokkei* and *Coomansus parvus* are indicated in bold. Numbers near nodes indicate posterior probabilities.

**Figure 10:**
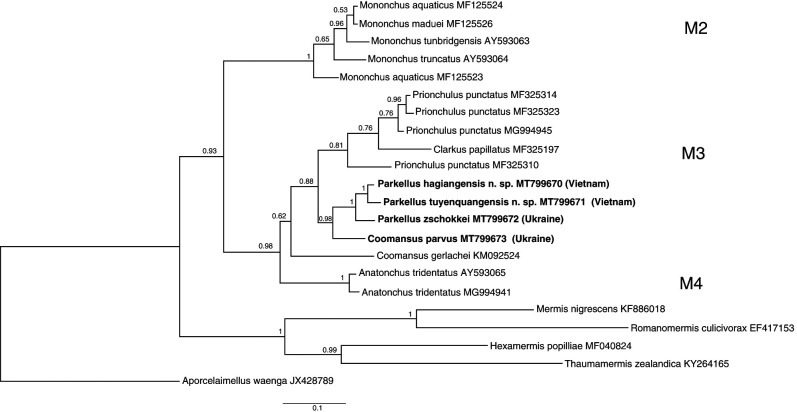
28S rDNA-based Bayesian phylogeny of the Mononchida. The new *Parkellus* species and the additionally studied *P. zschokkei* and *Coomansus parvus* are indicated in bold. Numbers near nodes indicate posterior probabilities.

Both, the 18S and 28S rDNA – derived sequences were acquired from 11 independent nematode individuals belonging to *P. zschokkei.* Three of the 18S rDNA sequences differed from the other eight in 1 extra nucleotide. All the acquired 28 rDNA sequences were identical. 18S rDNA: MT799667–MT799668 (approx. 1.6 kb); 28S rDNA: MT799672 (1 kb).The phylogenetic relationships of the acquired sequences are presented in Figures 9 and 10.

### Key to species of *Parkellus*

Currently, 12 species of the genus *Parkellus* have been recorded. The following key is modified from Loof and Winiszewska-Ślipińska (1993) and Ahmad and Jairajpuri (2010).

1.– Length of buccal cavity less than 61 µm............ 2

– Length of buccal cavity more than 65 µm......... 9

2.– Tail tip mucronate; gubernaculum thick, hooked proximally....................................... ***P. mucronatus***

– Tail tip without mucro; gubernaculum not hooked proximally.......................................................... 3

3.– Tail terminus slender, acute orsubacute............. 4

– Tail terminus conspicuously rounded................. 5

4.– Position of dorsal tooth apex 60–61% of buccal cavity length from its anterior end; *c′* = 3.5–4.0................................................. ***P. cobbi***

– Position of dorsal tooth apex 50–53% of buccal cavity length from its anterior end; *c′* = 2.5–3.4................................................ ***P. silvius***

5.– Tail in posterior third cylindroid with broadly rounded terminus; body slender (ratio *a* is close to 40)............................................. ***P. simmenensis***

– Tail uniformly narrowing with finely rounded terminus; body not so slender (ratio *a* is close to 30)..... 6

6.– Dorsal tooth located at the base of the vertical dorsal plate below the beginning of the pharynx, its apex position more than 71% of buccal cavity length from its anterior end........ ***P. hagiangensis***

– Dorsal tooth located at the base of the vertical dorsal plate above the beginning of the pharynx, its apex position not more than 70% of buccal cavity length from its anterior end......................... **7**

7.– Female tail 85–90 µm long; spicules 82–92 µm long, lateral guiding pieces with straight edges........................................................ ***P. parkus***

– Female tail 103–169 µm long; spicules 95–131 µm long, lateral guiding pieces with arched edges........................................................................ 8

8.– Position of dorsal tooth apex 60–70% of buccal cavity length from its anterior end; lateral guiding pieces 13–18 µm long; males with ventromedian cuticular pores above and below of the excretory pore.......... ***P***. ***tuyenquangensis***

– Position of dorsal tooth apex 47–60% of buccal cavity length from its anterior end; lateral guiding pieces 20–34 µm long; males without ventromedian cuticular pores above and below of the excretory pore........................ ***P. zschokkei***

9.– Body mostly well over 3 mm long; tail 150–190 µm long............................................ ***P. menzeli***

– Body mostly 2.0–2.7 mm long; tail 90–130 µm long.......................................................................... 10

10. – Tail slightly curved (about 40°); dorsal tooth rounded in contour.......... ***P. paraamphigonicus***

– Tail strongly curved (90° or more); dorsal tooth more or less pointed.............................................. 11

11. – Buccal cavity shorter (66 µm); tail tip acute. ..................................................... ***P. acuticaudatus***

– Buccal cavity longer (70–75 µm); tail tip rounded...................................................... ***P. monticola***

Analysis of interspecific sequence variation and phylogenetic relationships of the investigated *Parkellus* species

Molecular sequences of three *Parkellus* species were analyzed in this study: *P. zschokkei* from Ukraine, *P. hagiangensis* sp. nov. and *P. tuyenquangensis* sp. nov from Vietnam. These are the first molecular data available for the genus *Parkellus*. The interspecific nucleotide variation within the acquired 18S rDNA sequences was: *P. zschokkei* vs *P. tuyenquangensis* sp. nov. = 0.04*, P. zschokkei* vs *P. hagiangensis* sp. nov. = 0.02 and *P. tuyenquangensis* sp. nov. vs *P. hagiangensis* sp. nov. = 0.01. The interspecific 28S rDNA variation was: *P. zschokkei* vs *P. tuyenquangensis* sp. nov. = 0.05*, P. zschokkei* vs *P. hagiangensis* sp. nov. = 0.04 and *P. tuyenquangensis* sp. nov. vs *P. hagiangensis* sp. nov. = 0.02.

Three studied *Parkellus* species clustered together in a monophyletic group and were positioned within the M3 clade (following the nomenclature of Holterman et al., 2008), encompassing representatives of genera *Clarkus*, *Coomansus*, and *Prionchulus* (Figs. 9 and 10). This positioning was confirmed by the phylogenetic analyses based on both the 18S and 28S rDNA data. The close relationships of both Vietnamese species are also in agreement with their morphology. Their common morphological features are the lower position of the amphidial aperture and the medioventral pores above and below the excretory pore that are present only in males. However, the main feature that distinguishes two species from each other is the position of the dorsal tooth in relation to the beginning of the pharynx, in the area where it surrounds the base of the buccal cavity. Other differences were described in detail in the diagnosis part, in the description of *P. hagiangensis* sp. nov.

Our research delivers so far the only molecular data that are available for the genus *Parkellus* as well as one of the first ones belonging to the genus *Coomansus*. Inspite of this, there is still not enough sequences available in the data bases representing Mononchida species diversity. Accordingly, only three out of twelve *Parkellus* and only two out of 25 *Coomansus* species have been characterized molecularly (Vu, 2021). That is why it is too early, to definitely resolve the ongoing discussion, whether *Coomansus* and *Parkellus* belong to the same or different genera. At this moment, the comparison of the available 28S rDNA sequence diversity (only the 260 bp region as only a fragment of such length was available for *Clarkus*), the DNA distance between *Coomansus* and *Parkellus* are at the same level (3–5%) as the one between *Prionchulus* and *Clarkus*. Moreover, the *Coomansus gerlachei* species, currently represented only by the sequences deriving from the work of Elshishka et al. (2015), which do not cluster well within the M3 clade, should be more closely studied. Its phylogenetic positioning outside the ‘*zschokkei*-group’ was already mentioned by Mladenov et al. (2019) in their conference abstract. In conclusion, the frequently-raised question regarding the synonimisation of *Coomansus* and *Parkellus* should be investigated in more detail in further research on Mononchida.

## Conclusions

Two new species of the genus *Parkellus* from Vietnam, *Parkellus hagiangensis* sp. nov. and *Parkellus tuyenquangensis* sp. nov. are discovered based on the morphological and molecular characteristics. Their phylogenetic position in between Mononchida is fitted well within the M3 clade.
